# Patterns and motivations for method choices in suicidal thoughts and behaviour: qualitative content analysis of a large online survey

**DOI:** 10.1192/bjo.2021.15

**Published:** 2021-02-24

**Authors:** Lisa Marzano, Dafni Katsampa, Jay-Marie Mackenzie, Ian Kruger, Nazli El-Gharbawi, Denika Ffolkes-St-Helene, Hafswa Mohiddin, Bob Fields

**Affiliations:** Psychology Department, Middlesex University London, UK; Psychology Department, Middlesex University London, UK; Psychology Department, University of Westminster, UK; Department of Computer Science, Middlesex University London, UK; Psychology Department, Middlesex University London, UK; Psychology Department, Middlesex University London, UK; Psychology Department, Middlesex University London, UK; Department of Computer Science, Middlesex University London, UK

**Keywords:** Attempted suicide, suicide, self-harm, suicidal ideation, suicide method

## Abstract

**Background:**

Choice of suicide method can strongly influence the outcome of suicidal behaviour, and is an important aspect of the process and planning involved in a suicide attempt. Yet, the reasons why individuals consider, choose or discard particular methods are not well understood.

**Aims:**

This is the first study to explore method choices among people with a history of suicidal behaviour and individuals who have experienced, but not enacted, suicidal thoughts.

**Method:**

Via an online survey, we gathered open-ended data about choice of methods in relation to suicidal thoughts and behaviours, including reasons for and against specific means of harm.

**Results:**

A total of 712 respondents had attempted suicide, and a further 686 experienced suicidal thoughts (but not acted on them). Self-poisoning was the most commonly contemplated and used method of suicide, but most respondents had considered multiple methods. Method choices when contemplating suicide included a broader range of means than those used in actual attempts, and more unusual methods, particularly if perceived to be lethal, ‘easy’, quick, accessible and/or painless. Methods used in suicide attempts were, above all, described as having been accessible at the time, and were more commonly said to have been chosen impulsively. Key deterrents against the use of specific methods were the presence of and impact on other people, especially loved ones, and fears of injury and survival.

**Conclusions:**

Exploration of method choices can offer novel insights into the transition from suicidal ideation to behaviour. Results underscore the need for preventative measures to restrict access to means and delay impulsive behaviour.

## Background

Suicide is a leading cause of death worldwide.^1^ In England, the 2014 Adult Psychiatric Morbidity Survey found that a fifth of adults (20.6%) reported thoughts of taking their own life at some point, and one person in 15 (6.7%) has made a suicide attempt.^2^ Choice of method is one of the most important determinants of whether suicidal behaviour is fatal, and a key aspect of the process and planning involved in a suicide attempt. Indeed, earlier studies have shown that gathering information and access to means of suicide are known indicators of high suicidal intent and risk.^3,4^ Yet, previous literature has mostly focused on prevalence of methods, and associations with gender, age and mental disorder.^5^ How or why individuals consider, choose or discard particular methods are not well understood.

Epidemiological studies have shown that different methods, and combinations of methods, vary not only in relation to lethality, but also in the extent to which they predict subsequent suicidal behaviour and ‘method switching’.^6–10^ Their wider impact can also vary, not least by virtue of the exposure and potential clustering/‘contagion' effects associated with more public, unusual and ‘newsworthy’ methods.^11^ Indeed, avoiding ‘excessive detail of the method […] to prevent simulative acts’ remains to date the only official regulation for the reporting of suicide in the UK.^12^

This clearly points to the importance of better understanding the cultural and cognitive availability of different methods of suicide. Previous studies in this emerging field have identified these as key drivers in people's method choices, but have explored their significance in relation to a relatively narrow range of methods and with generally small study samples.^5^ Furthermore, earlier literature has tended to focus on fatal and non-fatal suicidal behaviour, overlooking its immediate, and generally less understood, precursor: suicidal ideation. Understanding the psychological processes preceding a suicide attempt is crucial for the potential to intervene, as is knowing what prevents the majority of individuals in distress from acting on thoughts of suicide, at a particular moment and/or over time.

The stability, or otherwise, of method choices also warrants further attention. A recent systematic review found that a third of individuals (33.3%) switch methods between successive episodes of self-harm, and almost half (42.11%) between an episode of self-harm and suicide.^13^ Based on prevalence of methods, there appears to be no discernible or predictable pattern to such means switching.^9,13^ Exploration of meanings, motivations and mechanisms may, however, offer valuable new insights. For example, a study in Austria showed that, despite method switching between episodes of self-harm being common, choice of means in the time immediately preceding a suicide attempt is often stable, and focused on a single method.^8^

## Aims

The present study aimed to investigate, for the first time, first-person accounts of the factors deterring and prompting consideration and/or use of specific methods of suicide among people with a history of suicidal behaviour and those who have experienced, but not enacted, suicidal thoughts. Dominant models of suicidal behaviour point to this as an important distinction, with potential for novel insights into the transition from suicidal ideation to behaviour.^14,15^ Given the disproportionate risk of suicide in men in the UK,^16^ gender differences in method choices were also explored, in relation to both suicidal ideation and behaviour.

## Method

### Online survey

Data were gathered as part of a wider study of method and location choices in relation to suicidal thoughts and attempts in the UK (QUEST, Qualitative Understanding of Experiencing Suicidal Thoughts^17,18^). A national online survey inviting people to share their experiences of suicidality was advertised through suicide prevention organisations such as Samaritans UK, online forums, social media and special interest groups. Study posters and leaflets were also placed on university bulletin boards, at local branch offices of relevant charities, in the National Suicide Prevention Alliance newsletter and were mailed out to supporters of the charity Campaign Against Living Miserably (CALM).

The 16-item anonymous survey asked participants if they had ever experienced suicidal thoughts and, if applicable, to describe in an open-text format whether this involved a specific method or methods, and why. Those who reported prior suicidal behaviour were then asked the same question in relation to their suicide attempt/s. All questions were optional, and no word limit, prompting or structure were imposed on open-ended responses. This also meant that multiple methods and/or reasons for (or against) using different means of suicide could be provided. Further information was gathered about the specific location or locations of suicidal thoughts and behaviours; about suggestions for preventative measures at different locations; and sociodemographic details (see Supplementary Appendix available at https://doi.org/10.1192/bjo.2021.15 for a copy of the full survey).

Links to further information about the study and to support services for those experiencing suicidal thoughts were available both at the beginning and the end of the survey. All participants gave informed consent to participate in this study, and all research materials and procedures were reviewed and approved by the Psychology Department Research Ethics Committee at Middlesex University (reference: ST019-2015).

### Statistical analysis

Open-ended survey responses were coded through a multi-stepped approach. Suicide methods were classified using the intentional self-harm (X60–X84) codes of ICD-10.^19^ Reasons for engaging in specific methods were analysed inductively for content,^20^ and an additional coding category was created to capture deterring factors, where reported. Three coders (D.F.St-H., H.M., N.E.-G.) coded 10% of the data, with substantial interrater reliability (Kraemer's kappa 0.774) (see Supplementary Appendix for a full description of the coding protocol and details of interrater reliability in relation to individual code categories).

Survey data are presented as frequencies or percentages, as appropriate. Variations in method choices were analysed using chi-square tests (for categorical variables) and *t*- and Mann–Whitney *U-*tests (for continuous variables). All statistical analyses were performed at a 5% level of significance.

## Results

We analysed the responses of 1398 people (Table 1). Of these, 68.5% identified as females, 29.5% as males, and 2.0% as transgender/gender fluid. Participants had a median age of 32 years (range 16–73), and around a third were aged between 18 and 30 years old (37.7%). The majority of the participants described themselves as White (92.8%), heterosexual (74.7%), and non-religious (53.0%).

**Table 1:** Self-reported characteristics of the participants

	All participants (*n* = 1398)	Prior suicidal ideation only (*n* = 686)	Prior suicidal behaviour (*n* = 712)
Age, years: median (range), *n*	32 (16–73) 1282	32 (16–73) 606	32 (16–72) 676
<18 years old, *n* (%)	120 (9.4)	61 (10.1)	59 (8.7)
18–30 years old, *n* (%)	483 (37.7)	224 (37.0)	259 (38.3)
30–45 years old, *n* (%)	436 (34.0)	201 (33.2)	235 (34.8)
45–60 years old, *n* (%)	214 (16.7)	102 (16.8)	112 (16.6)
>60 years old, *n* (%)	29 (2.3)	18 (3.0)	11 (1.6)
Gender, *n* (%)			
Female	896/1308 (68.5)	390/618 (63.1)*	506/690 (73.3)*
Male	386/1308 (29.5)	215/618 (34.8)	171/690 (24.8)
Transgender/gender fluid	26/1308 (2.0)	13/618 (2.1)	13/690 (1.9)
Sexual orientation, *n* (%)			
Heterosexual	923/1235 (74.7)	457/591 (77.3)	466/644 (72.4)
Bisexual	167/1235 (13.5)	69/591 (11.7)	98/644 (15.2)
Gay/lesbian	106/1235 (8.6)	48/591 (8.1)	58/644 (9.0)
Ambivalent/unsure	19/1235 (1.5)	7/591 (1.2)	12/644 (1.9)
Asexual	20/1235 (1.6)	10/591 (1.7)	10/644 (1.6)
Any religion (versus none), *n* (%)	572/1217 (47.0)	262/581 (45.1)	310/637 (48.7)
Ethnicity, *n* (%)			
White	1147/1236 (92.8)	548/588 (93.4)	599/648 (92.4)
Asian	47/1236 (3.8)	24/588 (4.1)	23/648 (3.5)
Black and minority ethnic	14/1236 (1.1)	3/588 (0.1)	11/648 (1.7)
Mixed race	28/1236 (2.3)	13/588 (2.2)	15/648 (2.3)

Denominators vary because of missing data.

* χ^2^ = 16.12; *P* < 0.001.

All participants reported prior thoughts of suicide. Of these, 686 respondents (49.1%) had experienced suicidal ideation but not engaged in suicidal behaviour; 712 (50.9%) reported having engaged in at least one suicide attempt. The latter subsample included a higher proportion of women, but did not differ significantly from the former in relation to the other sociodemographic characteristics captured (Table 1).

### Self-reported method choices: suicidal thoughts

When asked about previous thoughts of suicide, all but a small minority of respondents reported having considered a specific method, or methods (*n* = 1279, 91.5%). For most (*n* = 799, 57.2%), multiple methods had been contemplated (median 2; maximum 10), at different times or over time. On average, respondents with a prior history of suicidal behaviour reported having considered more suicide methods than those who had never attempted suicide (mean 2.17 (s.d. = 1.48) *v.* 1.84 (s.d. = 1.3), *P* < 0.001), with no significant difference between male and female respondents.

Overall, self-poisoning was the most frequently contemplated method of suicide (809/1398, 57.9%), including when only one method was considered (220/480, 45.8%). Approximately a quarter of the participants had experienced thoughts of suicide by hanging (381, 27.3%) or jumping from a high place (330, 23.6%) and fewer had considered self-harm by a sharp object (275, 19.7%), train (208, 14.9%) or other vehicle collision (208, 14.9%; all other methods were mentioned by under 10% of the participants).

There were some significant differences in the suicide methods contemplated by those who had and had not engaged in suicidal behaviour. The latter were more likely to cite relatively unusual methods such as crashing a vehicle (thoughts only versus behaviour: 85/686 (12.4%) *v*. 41/712 (5.8%); χ^2^ = 18.74; *P* < 0.001) and handgun discharge (thoughts only versus behaviour: 36/686 (5.2%) *v*. 21/712 (2.9%); χ^2^ = 4.72; *P* = 0.030), whereas those with a history of suicidal behaviour were more likely to report thoughts of suicide by hanging, strangulation or suffocation (behaviour versus thoughts only: 229/712 (32.2%) *v*. 153/686 (22.3%); χ^2^ = 17.10; *P* < 0.001), self-poisoning (behaviour versus thoughts only: 489/712 (68.7%) *v*. 320/686 (46.6%) χ^2^ = 69.56; *P* < 0.001) and self-harm by a sharp object (behaviour versus thoughts only: 159/712 (22.3%) *v*. 116/686 (16.9%); χ^2^ = 6.50; *P* = 0.011).

Self-poisoning and self-cutting were more commonly reported by female respondents, compared with males (self-poisoning, females versus males: 606/896 (67.6%) *v*. 151/386 (39.1%); χ^2^ = 90.7, *P* < 0.001; self-cutting, females versus males: 193 (21.5%) *v*. 60 (15.5%); χ^2^ = 6.1, *P* = 0.013). In contrast, male respondents were more likely to have considered death by hanging, strangulation and suffocation (males versus females: 131 (33.9%) *v*. 221 (24.7%); χ^2^ = 11.65; *P* = 0.001) and, but less frequently, by gasses and vapours (males versus females: 27 (7.0%) *v*. 31 (3.5%); χ^2^ = 7.81; *P* = 0.005), or handgun discharge (males versus females: 30 (7.8%) *v*. 24 (2.7%); χ^2^ = 17.35; *P* < 0.001).

### Suicidal behaviour and method choice ‘switching’

Fewer, and often different, methods choices were described in relation to actual suicide attempts (median number of methods reported in relation to suicidal behaviour  1; maximum 7). Self-poisoning was again the most commonly reported method in this context (555/712, 77.9%), particularly among female respondents who had attempted suicide (females versus males: 426/506 (84.2%) *v.* 104/171 (60.8%), χ^2^ = 41.1, *P* < 0.0001). This was followed by self-harm by sharp object (118/712, 16.6%), hanging (103/712, 14.5%; males versus females: 37 (21.6%) *v*. 64 (12.6%); χ^2^ = 8.1, *P* = 0.004), and jumping from a high place (60/712, 8.4%).

A small proportion of those who had considered self-poisoning when contemplating suicide (47/602, 7.8%) reported having adopted a different method or methods when engaging in suicidal behaviour. Relatively less common methods, such as jumping/falling from a height, drowning or vehicle collision, were reported considerably more frequently in relation to thoughts than actual suicidal behaviour.

Among respondents who had made one or more suicide attempts, the vast majority who had considered jumping/falling from a height (137/197, 69.5%), or in front of a train (96/116, 82.8%) or other vehicle (54/86, 62.8%), had not then attempted suicide by these specific methods. This was also observed in relation to suicide by gasses and vapours (24/33,72.7%), chemicals and noxious substances (28/45, 62.2%), drowning (52/77, 67.5%), crashing of a motor vehicle (38/48, 79.2%), and less frequently reported methods such as handgun discharge (17/22, 77.3%), and smoke, fire and flames (7/10, 70%). A smaller, but considerable, proportion of those who had contemplated suicide by hanging (143/246, 58.1%), sharp object (91/209, 43.5%) or poisoning by alcohol (29/84, 34.5%) had also then used different methods when attempting suicide.

### Motivations for and against method choices

Self-reported reasons for considering one or more methods of suicide were varied and often multiple. In describing their motivations for contemplating or attempting suicide by a specific method or methods, most respondents focused on more than one factor, particularly when discussing suicidal thoughts (815/1238, 65.8%, maximum 11; 359/679, 52.9% mentioned more than one reason in relation to suicide attempts, maximum 8).

**Fig. 1:**
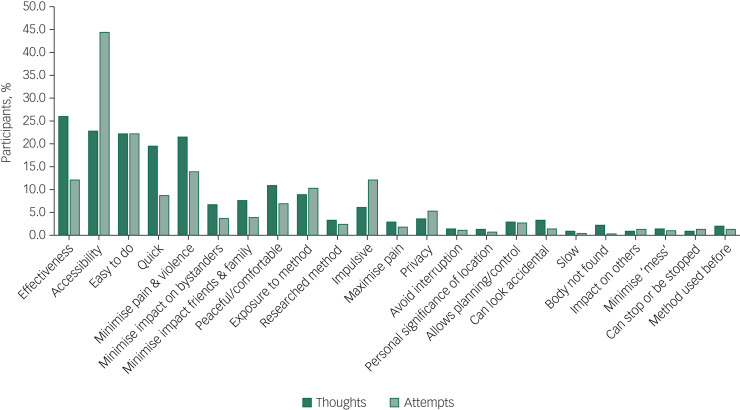
Self-reported motivations for method choices in relation to suicidal thoughts (*n* = 1398) and suicide attempts (*n* = 712).

There were some interesting differences in the reasons given for considering a specific method when contemplating suicide, as opposed to engaging in suicidal behaviour (Fig. 1).

Effectiveness, accessibility, ease and speed of a suicide method were the most commonly cited reasons for contemplating suicide by a specific method, alongside the wish to minimise pain and violence. Almost 70% of participants who stated one main reason for contemplating suicide by specific means cited one of these factors (287/423, 67.8%). However, when respondents commented on their motivations for attempting suicide by a given method, accessibility became the single most recurrent concern (reasons for suicidal behaviour versus ideation (all participants): 316/712 (44.4%) *v*. 319/1398 (22.8%), χ^2^ = 104.3, *P* < 0.0001), and impulsivity a more frequently cited reason (behaviour versus ideation: 86/712 (12.1%) *v*. 85/1398 (6.1%), χ^2^ = 22.8, *P* < 0.0001), particularly among women (females versus males: 70/506 (13.8%) *v*. 12/171 (7%), χ^2^ = 5.6, *P* = 0.018).

Accessibility was the most commonly reported reason for attempting suicide by a specific method even when excluding motivations for self-poisoning (the most common method of suicidal behaviour in the participants), and in relation to each method individually (alongside method ease and/or effectiveness for attempts by chemicals and noxious substances, other gasses and vapours, and handgun discharge). The only exceptions were attempts by jumping or lying in front of a vehicle (including trains), for which method effectiveness was the most frequently cited reason (13/45, 28.9%), and smoke, fire and flames (for which, however, only three participants provided data).

When discussing suicidal behaviour, ease of method remained among the more frequently cited reasons for choosing a specific method, but speed, effectiveness and the desire to minimise pain and violence were less commonly mentioned than in relation to suicidal thoughts (speed, behaviour versus ideation: 62/712 (8.7%) *v*. 272/1398 (19.5%), χ^2^ = 39.4, *P* < 0.0001; effectiveness, behaviour versus ideation: 86/712 (12.1%) *v*. 364/1398 (26.0%), χ^2^ = 54.8, *P* < 0.0001; minimising pain and violence, behaviour versus ideation: 99/712 (13.9%) *v*. 300/1398 (21.5%), χ^2^ = 17.6, *P* < 0.0001).

Among those who had engaged in suicidal behaviour, effectiveness appeared to be a marginally (but significantly) greater concern for males (males versus females: 29/171 (17.0%) *v*. 54/506 (10.7%), χ^2^ = 4.7, *P* = 0.03) and reducing pain and violence for females (females versus males: 82/506 (16.2%) *v*. 14/171 (8.2%), χ^2^ = 6.8, *P* = 0.009). In total, 10% of respondents reported having used a specific method of suicide following exposure to that method (via family and friends as well as media reports). Less common reasons, in relation to both suicidal thoughts and behaviour, included a desire for privacy and to avoid interruption, to minimise the impact on loved ones and other bystanders, or to maximise pain.

Some of the differences in the decision-making around suicidal thoughts versus behaviour were reflected in the motivations for contemplating a specific method in those who had engaged in suicidal behaviour and those who had not. In particular, accessibility of method was more frequently mentioned by those with a history of suicidal behaviour (behaviour versus thoughts only: 180/712 (25.3%) *v*. 139/686 (20.3%), χ^2^ = 5.0, *P* = 0.025), alongside perceived lethality (behaviour versus thoughts only: 205/712 (28.8%) *v*. 159/686 (23.2%), χ^2^ = 5.7, *P* = 0.017) and, less often, a wish to maximise pain (behaviour versus thoughts only: 32/712 (4.5%) *v*. 9/686 (1.3%), χ^2^ = 12.41, *P* < 0.0001). Respondents who had never acted on suicidal thoughts were instead more likely to mention wanting to minimise the impact on others (thoughts only versus behaviour: 116/686 (16.9%) *v*. 83/712 (11.7%), χ^2^ = 7.9, *P* = 0.005), including and especially family and friends (thoughts only versus behaviour: 63/686 (9.2%) *v*. 43/712 (6.0%), χ^2^ = 4.9, *P* = 0.026), and, in smaller numbers, to be in an isolated location where one's attempt and body would not be discovered (thoughts only versus behaviour: 21/686 (3.1%) *v*. 10/712 (1.4%), χ^2^ = 4.4, *P* = 0.035).

Concern about the impact on others was also a key theme in the responses of participants who mentioned motivations for not using specific methods (57 respondents discussed discarding a particular method of suicide for this reason), alongside fears around survival (potentially with injuries, *n* = 58), and of not ‘getting it right’ (*n* = 51). Further deterring factors included concerns over the violent/painful nature of a method (*n* = 35), its ‘messiness’ (*n* = 11) or slowness (*n* = 7), lack of access (*n* = 19) or privacy (*n* = 5), and the discarding of a method having used it previously (*n* = 4) or researched it (*n* = 9).

## Discussion

### Main findings and interpretation

Although specific methods, and composite methods, have been associated with a higher risk of death and subsequent suicidal behaviour, epidemiological analyses of repeat hospital admissions for self-harm have concluded that ‘method of self-harm is fluctuating and unpredictable’.^9^ The results of this exploratory study of first-person accounts suggest that, although indeed changing and complex, method choices are reasoned, personally and culturally meaningful, and a crucial element of the processes and planning involved in attempting (or desisting from) suicide. With very few exceptions, individuals who had contemplated suicide had considered a specific mean (or, more often, means) of taking their own life, even when they had never then engaged in suicidal behaviour. Almost 90% described a particular reason, or set of reasons, for considering or discarding such method/s.

To date, method choices have primarily been investigated in relation to completed suicide and suicide attempts.^5,21,22^ This is the first study to explore patterns and motivations for method choices in relation also to suicidal ideation, which is a surprising gap in knowledge given that 10–20% of individuals experiences lifetime suicidal ideation.^2,23^ Suicidal thoughts are a known risk factor and immediate precursor of suicidal behaviour, and key to understanding the transition from ‘suicidal ideation to action’.^14,15^ In relation specifically to methods choices, suicidal thoughts provide some important insights into the acceptability and cognitive availability of different means of suicide. In other words, they can help us understand which methods are cognitively available to individuals in crisis (in a given sociocultural context), and what makes them more or less ‘attractive’ to them.

In this study, self-poisoning was the most commonly contemplated method of suicide. However, most respondents had considered multiple methods of suicide, particularly those who had also engaged in suicidal behaviour. These included a fairly wide range of potential methods and, especially among individuals who had never attempted suicide, relatively uncommon means such as jumping or lying in front of a moving object, drowning, handgun discharge and crashing of a motor vehicle. In England and Wales, self-harm involving a moving object, drowning or fall and fracture accounts, all together, for 12% of all suicides,^24^ and only 1% of hospital presentations for self-harm.^10^ Firearm use, although a common suicide method in countries with less stringent gun control such as the USA,^25^ is even rarer in the UK, accounting for less than 2% of all suicides^26^ and 0.03% of self-harm hospital presentations.^10^ Although these are only a small proportion of completed suicides and hospital presentations for self-harm, such methods tend to be disproportionately reported in the media,^11^ which may reflect and account for their over-representation and over-availability in people's suicidal thoughts (in the UK, most suicides are hanging^16^ whereas most hospital presentations for self-harm involve self-poisoning^10^). Almost 10% of the participants explicitly identified exposure to a given method as a primary motivation for considering it as a means of suicide. Even more common reasons were perceived method ease, accessibility, speed and effectiveness, alongside the desire to minimise pain and violence.

However, the range of methods described in relation to actual suicidal behaviour was narrower, as were respondents’ motivations for using such methods. Over three quarters of respondents attempted suicide by self-poisoning, with many switching to this method having also considered more violent or unusual means. For almost half of the participants, choice of attempt method was primarily dictated by accessibility, and ease of method and the minimisation of pain were more common concerns than perceived speed or likelihood of death. An exception was jumping/lying in front of a vehicle, for which method effectiveness was the most frequently cited reason (see also Marzano et al^18^).

In relation to all methods, lethality appeared to be a greater concern for male than female respondents, and impulsivity less common, as also reported elsewhere.^27,28^ Together with the increased use of methods other than self-poisoning,^29^ this might contribute to the disproportionate risk of suicide in men.

Of note are also some of the differences between respondents who had contemplated but never engaged in suicidal behaviour, and those who had previously attempted suicide. The former were more likely to mention wanting to minimise the impact on others, especially family and friends, and, in smaller numbers, to be in an isolated location where one's attempt and body would not be discovered. This points to the presence and impact on bystanders and loved ones as important ‘dissuaders’ in relation to suicide. Indeed, these were also among the most frequent reasons for not attempting suicide by a specific method. Other common deterrents were fears of surviving with injuries and of ‘not getting it right’.

Interestingly, impulsivity was not a more common theme in those who had attempted suicide *per se*, but was cited more frequently in relation to suicidal behaviour than ideation. This finding lends support to the idea that individuals who attempt suicide may not have significantly elevated trait impulsiveness, compared with ‘ideators’; however, they may have higher impulsiveness when in a negative state.^30^

### Strengths and limitations

We analysed rich descriptions of people's choice of methods in relation to suicidal thoughts as well as behaviours, with strong interrater reliability, and no prompting, structure or limit to the answers that could be provided. This exploratory, inductive approach is rare with samples as large as the current study's, and allows for appropriately powered statistical analyses, as well as more nuanced, in-depth analyses of particular groups or methods, including the different images, myths and cultural scripts that exists around – and against – specific means of suicide (as presented elsewhere in relation to railway suicide^18^).

However, findings were based on a self-selected, predominantly female sample, and may not necessarily be representative of all individuals who consider, attempt or indeed die by suicide, within the UK and more widely. National and cultural variations in suicide method choices suggest the need for replication with broader and more diverse samples,^25^ in a wider range of community and clinical settings.

Given the exploratory nature of this study, we did not gather systematic information about potentially important factors such as prior psychiatric and family history, nor about the sequence or temporality of suicidal thoughts and behaviour. This limits the conclusions that may be drawn in relation to method switching, escalation and substitution, and prevented us from making inferences about age differences in method choice. Further, longitudinal studies could usefully investigate how the frequency, intensity and intent of suicidal thoughts and behaviour may affect method choices in different groups and communities. A more structured approach to gathering information about deterring factors could also offer important insights into how best to prevent, and ‘dissuade’ from, specific means of suicide, in different contexts and locations.

### Implications

Previous research has concluded that ‘people's risks or needs [cannot be] based simply on the method of harm’,^9^ or the potential lethality of that method. The results of this study also suggest that the methods people consider when contemplating suicide are often not the ones that are then enacted in a suicide attempt, nor are they necessarily chosen for the same reasons. Nonetheless, exploring method choices is far from a fruitless activity, and can usefully inform preventative initiatives.

Consistent with earlier literature, our findings suggest that cognitive and physical availability are key drivers in the choice and prevalence of suicide methods. The former appears to be particularly relevant in the context of people's suicidal thoughts, with methods perceived to be ‘easy’ and lethal featuring highly in individuals’ so called ‘ideation menu’.^31^ The latter (i.e. the accessibility of a given method), becomes especially important in the context of a suicide attempt, and impulsivity relatively more common. This clearly underscores the need for preventative measures that restrict access to means and delay impulsive behaviour, such as the erection of barriers at high-risk locations and reduced pack sizes of paracetamol.^32^ Avoiding depictions and descriptions of suicide methods in the media may help limit the cognitive availability of specific means, particularly for methods portrayed as lethal, easy, quick and painless. This may be especially important in the reporting of celebrity suicides. A recent meta-analysis found that when the suicide method used by a celebrity was reported, there was an associated 30% increase in deaths by the same method.^33^

In clinical settings, exploring the decision-making around specific methods may help challenge unhelpful myths and misconceptions, identify areas of ambivalence and hope, and develop appropriate safety and treatment plans. At public-health level, factors known to attract individuals and subgroups to lethal means of suicide may be challenged via targeted communication and media strategies, and common ‘dissuaders’ reinforced. However, the risk and unintended consequences of different approaches to means and ‘myth-restriction’ need careful thought and evaluation, whether in the context of means-restriction counselling^34^ or as part of wider public health approaches. Further research is needed in this area, but the risks and ethics of how and where research findings are disseminated and ‘translated’ need careful attention to avoid reinforcing the acceptability and availability of (lethal) means of self-harm.

## Data Availability

The data that support the findings of this study are available on request from the corresponding author (L.M.). The raw data are not publicly available as they include qualitative quotes that could compromise the privacy of research participants.

## Supplementary material

For supplementary material accompanying this paper visit http://dx.doi.org/10.1192/bjo.2021.15. click here to view supplementary material
