# Supplementary material for: Patterns and motivations for method choices in suicidal thoughts and behaviour: qualitative content analysis of a large online survey
Source: BJPsych Open. 2021 Feb 24;7(2):e60. doi: 10.1192/bjo.2021.15 (PMC8058851; doi:10.1192/bjo.2021.15)
Supplement: Supplementary file 1 [file S2056472421000156sup001.docx]

**Supplementary materials for “Patterns and motivations for method choices in suicidal thoughts and behaviour: Qualitative content analysis of a large online survey”**

***Content Analysis of Survey Responses: A multi-stage approach***

The online survey aimed to explore respondents’ suicidal thoughts and attempts (please see Appendix 1 for a full copy of the online survey). Firstly, by asking if they had experienced suicidal thoughts and if so whether these thoughts involved a particular method or methods, and then to describe in an open text format what these were. A second open text question asked them to expand on why they chose these methods by asking what influenced their decision to use the method(s). Next respondents were asked if their suicidal thoughts involved a particular location, whether this was a private or public place, and if a public place whether it was a train/tube station or railway, public building; public road or bridge; park or country side; or other (with an option to describe what was meant by ‘other’). An open-ended question followed asking why this particular location or locations was chosen, and then what participants thought could be done to prevent suicide a this/these or similar locations. A second block of questions followed the same format, but in relation to suicide attempts. At the end of the survey, participants answered questions (also open-ended) about key socio-demographic characteristics, and had the opportunity to record any further comments or suggestion in an open text box.

Analysis of these data was beset by two interrelated problems: the first is that respondents gave multiple answers within one question, so for example the method question would typically result in a list of several. Secondly, there was a good deal of overlap in content among the question responses (for example respondents would answer question by referring to and/or reiterating what they had written in previous questions).

Processes had to be developed in order to deal with the complexity of the resultant data. In particular, we followed a multi-stepped approach:

**1. Text analysis**: The first step was to do a text analysis of each of the responses using SPSS Text Analytics for Surveys tool, producing a list of concepts which were then built up and restructured to create a suicide specific thesaurus. In turn, this provided a starting point for a set of codes.

Another useful starting point that shaped the codes for suicide methods was the International Classification of Diseases, which provides a classification of intentional self-harm (X60-X84). The codes for suicide method were closely based on ICD-10 (<http://apps.who.int/classifications/icd10/browse/2016/en>).

In addition, an iteration of thematic coding on the reasons for choosing method(s) had been done by two researchers working on the interviews, which contributed to the collection of concepts under themes, renaming and combining the text analysis concepts further. This finally produced an initial list of codes with associated words and phrases that coders in the second phase could expect to find in the responses as evidence for assigning a particular code to a response.

**Limitations of text analysis:** The text analysis was limited on several levels. Firstly, it was not possible to develop the dictionary beyond a very basic level of a collection of words and phrases. Secondly, text analysis, for all its power, cannot provide the necessary context to the words categorised under specific themes. It was therefore necessary to have another layer in the analysis of the data and treat the text analysis as a source for initial codes and as partial definition of the codes.

**2. Coding of complete responses with a web-based coding tool:** In order to present the respondents’ entire response and support the coding process, an online tool was created, as shown in Figure 1. The collection of data could be filtered by keywords and other criteria (Data Filtering and Selection area), and the content of a single response viewed (Questions and Responses area). The coder was thus able to view the questions and the answers given by a respondent, and make decisions about how the response should be coded. Possible codes were available as menu selections (Coding Choices area), so that the coder could assign codes in the context of the respondent’s entire response (including the comments section at the end of the survey). This was necessary because respondents often referred to what they had written in previous questions and in each question multiple answer were frequently given. Next to each response, the relevant codes were repeated, as shown below.


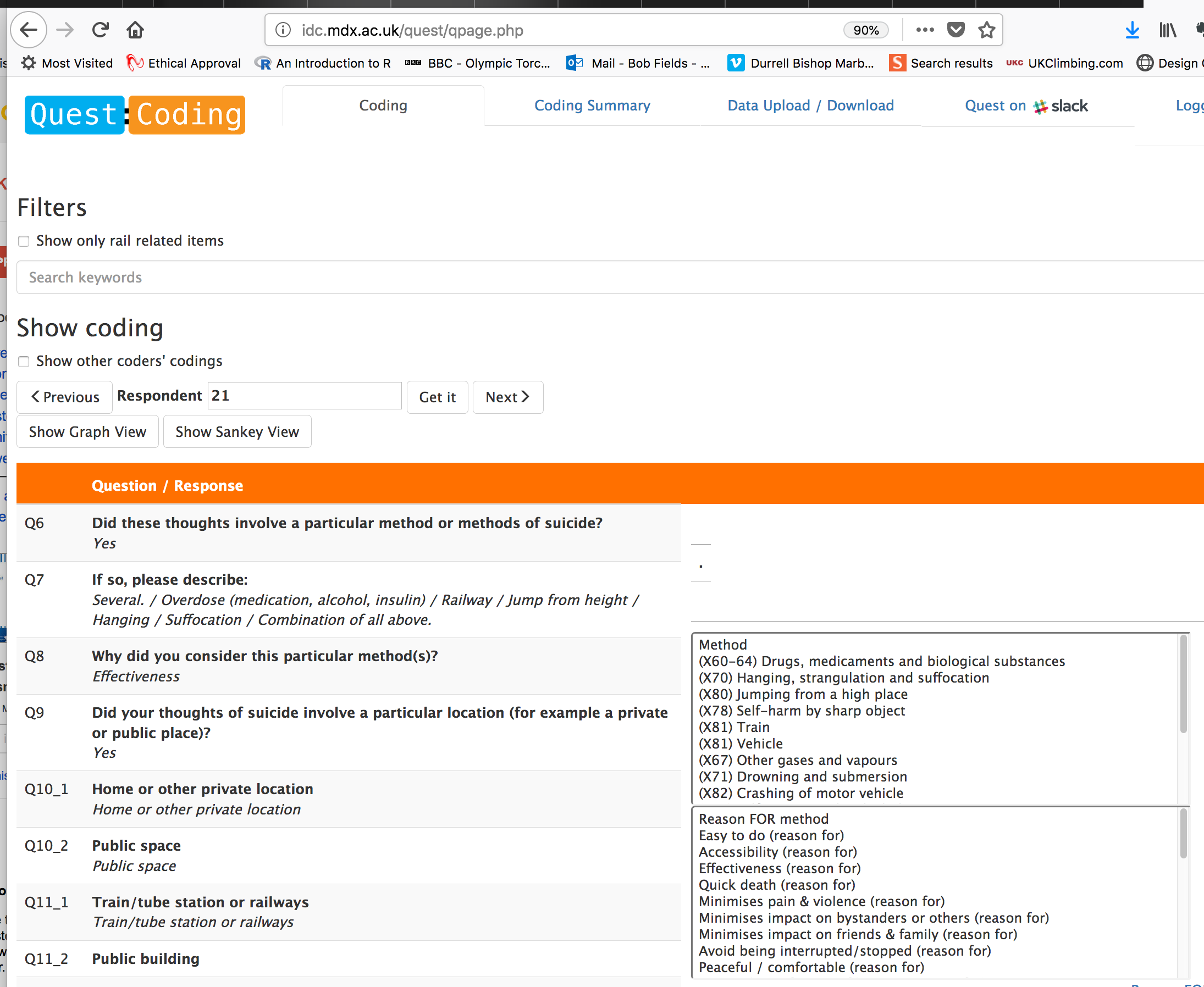


Questions and Responses

Coding Choices

Data Filtering and Selection

Figure 1 Layout of online coding tool, with three key areas highlighted

***Coding protocol.*** A coding protocol was developed, in relation to 6 main code categories (see Figure 3), applied to suicidal thoughts first and then suicide attempts (where reported).

Guided by the main line of questions, coders would identify and code

- A method (1)
- Reason(s) for or reasons against this method, if evident (2).
- The location (3)
- Reason(s) for that location (4)
- Finally, possible prevention measures (5).

Together, this identification of codes for Method, Reasons, Locations, Reasons and Preventions constituted a “coding thread” (a “thread” through the respondent’s story). Coding of a response was exhausted when all methods had been identified. Evidence for these codes could be found in any part of the response, although there was a separate set of codes for attempts and coders were asked to separate out what was clearly associated with an attempt from what were just thoughts, if possible. A response could contain several “threads” if the respondent has described contemplating or attempting to use several different methods.


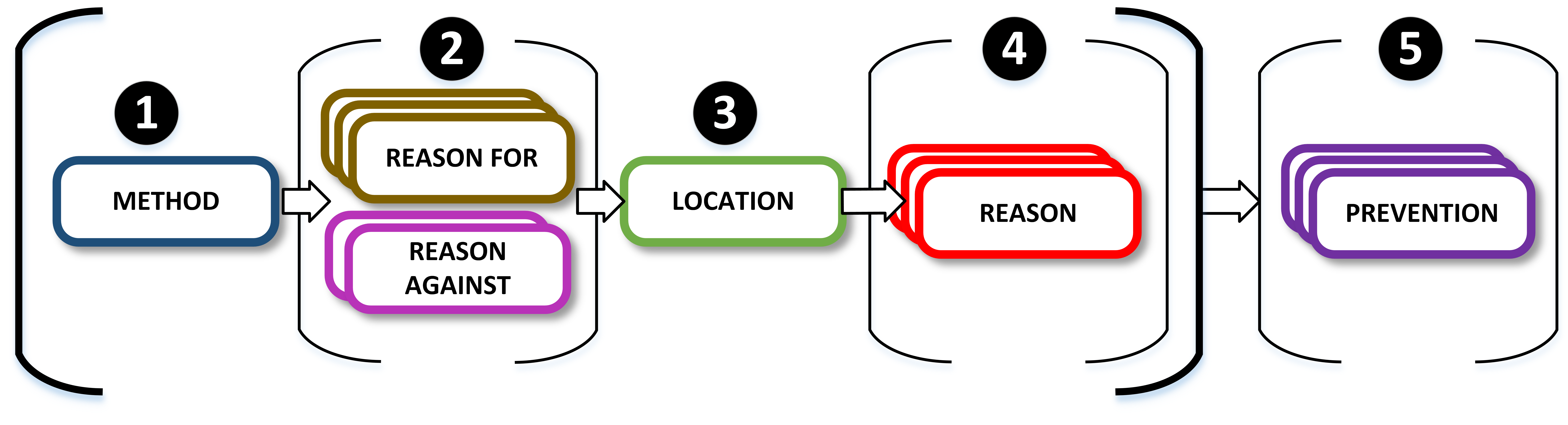


Figure 2 Coding Protocol

Table 1 illustrates the process with the content of one response, along with two of the threads identified by coders.

After a response has been coded, each of the threads generated could be displayed in both textual and graphical form for review. If more than one coder had coded the same response, this visualization could help to identify disagreements and potential divergences in understanding, either of the data or of the coding scheme, that led to the disagreement. Figure 3 shows the graphical visualization of the threads generated by several coders.

Table 1 Example Response and Coding Threads

| Response number 21 | |
| --- | --- |
| Q6. Did these thoughts involve a particular method or methods of suicide? | Yes |
| Q7. If so, please describe: | Several. / Overdose (medication, alcohol, insulin) / Railway / Jump from height / Hanging / Suffocation / Combination of all above. |
| Q8. Why did you consider this particular method(s)? | Effectiveness |
| Q9. Did your thoughts of suicide involve a particular location (for example a private or public place)? | Yes |
| Q10_1. Home or other private location | Yes |
| Q10_2. Public space | Yes |
| Q11_1. Train/tube station or railways | Yes |
| Q11_2. Public building |  |
| Q11_3. Public road or bridge | Yes |
| Q11_4. Park or countryside | Yes |
| Q11_5. Other | Yes |
| Q11_5. Other-TEXT | Mountains |
| Q12. Why did you consider this particular location or locations? | Effectiveness / Isolation / |
| Q13. What do you think could be done to help prevent suicide at this or similar locations? | Barriers, cctv, support contacts |
| Q30. Additional comments | Multiple attempts aged 9, 14, 17 / Thoughts and plans permanent since then. / Only thing that stops me is knowing that no method certain to work and I will not fail again |

| Thread A |  |
| --- | --- |
| Method: | (X81) Train |
| Reasons for: | Effectiveness (reason for) |
| Location: | Train/tube station or railways |
| Reason for location: | Effectiveness (reason for) \| Privacy (reason for) |
| Prevention: | barriers, fences, railings; CCTV; people to talk to |

| Thread B |  |
| --- | --- |
| Method: | (X80) Jumping from a high place |
| Reasons for: | Effectiveness (reason for) |
| Location: | Public road or bridge |
| Reason for location: | Effectiveness (reason for) |
| Prevention: | signs and posters; barriers, fences, railings; CCTV |


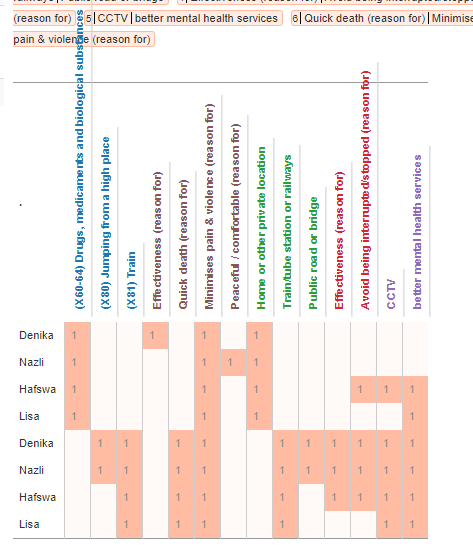


Figure 3. Graphic display of all coders' thread

Development of the codes. The codes were developed by refining the definitions where subtleties were encountered in the full text. Secondly, any response that did not fit the coding scheme was given a new code which was then available as an option for further coding. It is important to note that reasons for location were given as a repeat of the method reasons but with expectation that each code would be treated as referring to the qualities of the location.

***Coding practice.*** Three coders applied this coding framework to an initial common set of responses. Discussions around the coded responses helped refined the codes based on their applicability, and develop consensus on their interpretation and applicability. Once confidence in the codes and in a common understanding of their applicability had been established amongst the coders, each coded a section of those responses that contained rail related responses. The rail related responses were identified through key words and phrases from the Methods dictionary.

***Wiki development.*** A shared online space was created to help build up a commonly understood and robust set of definitions for the codes. This included an operational definition of all codes used in the analysis, which are available at <http://idc.mdx.ac.uk/quest/codingwiki>.

***Inter-rater reliability.*** In order to assess the robustness of the coding scheme and coding practices, and to diagnose any problems or disagreements that arose during coding, around 10% of the responses were coded by all three coders. Inter-rater reliability was assessed by computing Kraemer’s extension of the kappa statistic^1^. This way of calculating kappa is applicable in situations where each item may be assigned multiple codes by multiple coders. Across all coding categories, the calculated value of kappa was 0.774, a level typically judged to be a ‘substantial’ or ‘excellent’ level of agreement.

In an attempt to reveal where problems and coding disagreements may be occurring, the kappa statistic was computed separately for each code category, as shown in Table 2. A high level of agreement can be seen across most of the categories, with the only coding in the ‘Reasons AGAINST method’ (a rather small category) and ‘Reasons for location’ categories showing a level of agreement that is ‘fair to good’:

Table 2: Inter-rater reliability across coding categories showing Kraemer’s kappa and the number of responses coded in each category

| Coding category | Kappa | N |
| --- | --- | --- |
| Method | 0.904 | 120 |
| Reason FOR method | 0.774 | 118 |
| Reason AGAINST | 0.514 | 22 |
| Location | 0.787 | 108 |
| Reason for location | 0.677 | 102 |
| Prevention | 0.723 | 98 |

**References**

1. Kraemer HC. Extension of the kappa coefficient. *Biometrics*. 1980. doi:10.2307/2529972.

**APPENDIX 1: ONLINE SURVEY**

**QUEST – Qualitative Understanding of Experiencing Suicidal Thoughts**

***Below are a series of questions about your experiences of suicidal thoughts. When answering these questions, please focus on your own personal thoughts and experiences, not what might be true in general, or for other people. There are no right or wrong answers; we are interested in what you think and what your own experience has been.***

**1. Have you ever had thoughts of ending your life?**

Yes (automatically go to Q2)

No (automatically go to Q5)

**2. Did these thoughts involve a specific method or methods of suicide?**

Yes (automatically go to next Q)

No (automatically go to Q3)

**If so, please describe:**

**2b. Why did you consider this particular method(s)?**

**3. Did your thoughts of suicide involve a particular location?**

Yes (automatically go to next Q)

No (automatically go to Q5)

If so, please tick as many as apply:

- Home or other private location
- Public place (automatically opens options below)

Please tick as many as apply:

- Train/tube station or railways
- Public building
- Public road or bridge
- Park or countryside
- Other: …………….

**3b. Why did you consider this particular location or locations?**

**4. What do you think could be done to help prevent suicide at this or similar locations?**

***The following questions ask about your experiences, if any, of attempting suicide.***

**5. Have you ever attempted suicide?**

Yes (aut. proceed below)

No (aut. go to socio-demographic Qs)

**6. If yes, what method or methods did this involve?**

**7. What do you think influenced your decision to use this particular method or methods?**

**8. Did your suicide attempt or attempts take place (please select as many as apply):**

- At home or in other private location
- In a public place (aut. opens options below)

Please tick as many as apply

- Train/tube station or railways
- Public building
- Public road or bridge
- Park or countryside
- Other: …

**9. What do you think influenced your decision to attempt suicide at this particular location or locations?**

**10. What, in your opinion, could help prevent suicide at this or similar locations?**

***Finally, to help us understand whether and how people’s experiences of suicidal thoughts vary across age, gender and other characteristics, we would be grateful if you could describe your:***

- Age:
- Gender:
- Sexual orientation:
- Ethnicity:
- Nationality:
- Religion (please state if not religious/atheist/agnostic):

***ADDITIONAL COMMENTS***

***Please use this space for any additional comments***
